# Evolution of the COVID-19 Pandemic: An Analysis of the Brunt of the Second and Third Waves on Patients in Western Uttar Pradesh

**DOI:** 10.7759/cureus.29251

**Published:** 2022-09-17

**Authors:** Prem P Mishra, Anil Kumar, Amit Garg, Priyanka Mahaur, Gunjan Bhatnagar, Deepak Upadhyay, Ramesh C Gupta, Ved Prakash

**Affiliations:** 1 Microbiology, Lala Lajpat Rai Memorial Medical College, Meerut, IND; 2 Community Medicine, Rohilkhand Medical College and Hospital, Bareilly, IND; 3 Ophthalmology, Lala Lajpat Rai Memorial Medical College, Meerut, IND; 4 Microbiology, Rohilkhand Medical College and Hospital, Bareilly, IND

**Keywords:** novel coronavirus, third wave, second wave, sars-cov-2, rtpcr, pandemic, covid-19

## Abstract

Background: The recent second wave and the latest third wave of coronavirus disease 2019 (COVID-19) in India caused havoc on health infrastructure. However, there is a scarcity of studies from India and abroad that compare the second and third waves of the COVID-19 pandemic. We aimed to assess the factors like age, sex, and death comparison among diagnostically proven COVID-19 patients of the Meerut district in both waves.

Methodology: A total of 297554 samples during the second wave (1^st^ March 2021 to 30^th ^June 2021) and 240655 during the third wave (1^st^ January 2022 to 30^th^ April 2022) were tested for reverse transcription polymerase chain reaction (RT-PCR) in the Department of Microbiology, Lala Lajpat Rai Medical College, using The Indian Council of Medical Research (ICMR) approved RT-PCR testing kits. The data like age, sex, place, follow-ups, etc. were recorded and data were analyzed statistically.

Results: The RT-PCR positivity of 8.24% for COVID-19 in the second wave while 5.66% of patients in the third wave have been reported. The proportion of positive cases in children ≤10 years in the second and third wave were quite similar i.e., 3.59% and 3.40% respectively, whereas the proportion of positive cases in adolescents (10-20 years) was significantly higher (12.96%) in the third wave in contrast to the second wave (10.15%), while age group (41-60 years) is significantly less (26.65%) in proportion during the third wave in comparison to the second wave (29.50%). The proportion of positivity in young males has significantly increased in the third wave as compared to the second wave. The mortality also decreased significantly by 1/3^rd^ of the second wave.

Conclusion: The third wave showed low overall positivity (5.66%) as compared to the second wave (8.24%), while the brunt on young children was comparable to the second wave which was assumed to be higher. The mortality and hospitalization also decreased significantly in the second wave. Regular surveillance and analysis should continue to combat this pandemic.

## Introduction

The first few cases of COVID-19 were recorded in December 2019, as cases of pneumonia in Wuhan, China, and then the World Health Organization (WHO) was informed about this as of unknown cause. On the 30^th^ of January 2020, WHO declared the hurriedly spreading COVID-19 outburst as a Public Health Emergency of International Concern, and on 11^th^ March 2020, COVID-19 was declared a pandemic. The first case of COVID-19 infection in India was reported in Thrissur, Kerala, India on January 27, 2020, who was a 20-year-old female with a one-day history of dry cough and sore throat [1].

The isolate on sequencing and phylogenetic analysis was confirmed and later named by the International Committee of Taxonomy of Viruses (ICTV) as severe acute respiratory syndrome coronavirus-2 (SARS-Cov-2), a novel etiological agent which belongs to the same family of SARS-CoV-1 and also was sequentially similar to bat derived Yunnan/RaTG13/2013 coronavirus strain [2]. Till 12^th^ July 2022, India recorded the second highest confirmed cases around the globe with 43,652,944 COVID-19 cases and the third highest death toll (525,474) due to COVID-19 infection [3,4]. The first wave of the COVID-19 pandemic commenced with increased detection of cases in January-March 2020, and after the September 2020 peak, cases declined by the end of October 2020 [5]. On the other hand, the second wave witnessed in India from April to May 2021 caused an unprecedented burden on the health sector of India contributing to nearly 47% of single-day incident cases around the globe during its peak [5]. Our country witnessed a low number of cases/day during the first wave but in contrast, the second wave witnessed more than 400,000 cases /day. The third wave of the pandemic was witnessed from January to March 2022; the new cases have increased exponentially during the phase with a high infectivity rate and low mortality rate and hospitalization.

The lineage analysis of the isolates in India showed the appearance of new variants of SARS-CoV-2, such as B.1.617.1 and B.1.617 which might be the reason for a sudden rise in the cases. The SARS-CoV-2 double mutant strain B.1.617, having the key structural mutations Glu484Gln and Leu452Arg in the spike protein, is highly infectious and less affected by the ongoing vaccine strategy [6,7]. The various variants of concern reported by WHO globally till date were Alpha B.1.1.7, Beta B.1.351, Gamma P.1, and Delta B.1.617.2 (mostly isolated in the second wave). Based on the genome sequencing the Omicron variant was the prime culprit behind the rapid but short-lived third wave. The lineage B.1.1.529 including BA.1, BA.2, BA.3, BA.4, and BA.5 was involved [8].

As the signs and symptoms of COVID- 19 are of a great array and both WHO and national case definitions have evolved with knowledge of COVID-19 etiology and the myriad of its manifestations [9,10]. It is imperative to diagnose a patient with SARS-CoV-2 based on laboratory testing. The laboratory diagnosis of a suspected COVID-19 patient includes a battery of tests: complete blood count (CBC), serum electrolytes, liver function tests (LFT), kidney function tests (KFT), prothrombin time (PT), international normalized ratio (INR), activated partial prothrombin time (aPTT), serum ferritin, C-reactive protein (CRP), lactate dehydrogenase (LDH), procalcitonin, plasma fibrinogen, D-dimer, arterial blood gases and most importantly radiological findings like X-ray, computed tomography (CT), magnetic resonance imaging (MRI). The strategy of laboratory testing is based on the detection of antigens and antibodies but the gold standard for diagnosis is the detection of viral nucleic acid by a real-time polymerase chain reaction in respiratory tract materials.

The previous data from different sources clearly shows that the second wave was much larger in magnitude, as compared to the first wave but very little information exists about the magnitude of the third wave and its demographic characteristics like age and sex differential. As various learned people thought that the pediatric age group will be more affected as compared to the adult age group in the third and subsequent waves if it comes. Hence, this study was undertaken to explicate the transformation in demographic factors among the confirmed cases of COVID-19 in the third wave.

## Materials and methods

Study design and period

The current study is a retrospective study in which data from 1^st^ March to 30^th^ June 2021 and 1^st^ January 2022 to 30^th^ April 2022 of laboratory tests carried out in the Department of Microbiology, Lala Lajpat Rai Memorial Medical College (LLRM Medical College), Meerut, were analyzed. The study was conducted after the approval of the Institutional Ethics Committee, LLRM Medical College, with approval number No./SC-1/2022/5127.

Sampling frame

As part of the COVID-19 surveillance program, 297554 samples for RTPCR testing during the second wave (1^st^ March 2021 to 30^th^ June) and 240655 during the third wave (1^st^ January 2022 to 30^th^ April 2022) were collected from all the centers in the Meerut district and tested (RTPCR) in Department of Microbiology, LLRM Medical College. The records of COVID-19 incident cases and related deaths were noted.

Inclusion and exclusion criteria

All confirmed cases of COVID-19 by RTPCR were included in the study. Samples other than RTPCR testing like antigen testing and radio-diagnostically proved COVID-19 cases were excluded. Samples without patient information sheet were not included.

Data collection

The data elements included age, sex, place, date of RTPCR testing, follow-up status, and deaths by SARS-CoV-2 infection were compiled.

Methodology

The nasopharyngeal and oropharyngeal swabs from the suspected samples were tested for RTPCR using the Covisure COVID-19 RTPCR kit (Genetix Biotech Asia, New Delhi, India); CoviPath COVID-19 RTPCR kit (Thermo Fisher Scientific, MA, USA), and Q line molecular nCov-19 RTPCR kit (Q-Line Biotech Pvt. Ltd, New Delhi, India). In some cases, repeat sampling was required due to poor quality of the specimen, inadequate sample, improper handling, and transportation of samples [11].

Data analysis

Statistical analysis of the data was performed using the SPSS statistics software package, version 11.0 (SPSS Inc., Chicago). Data were represented in tables and graphs wherever found appropriate. Data were expressed as means, median, etc. Comparisons between groups were performed with an analysis of the non-parametric test. A value of P < 0.05 was considered statistically significant.

## Results

During the study period of the second wave of the pandemic i.e., 1^st^ March 2021 to 30^th^ June 2021, a total of 297554 patients were tested by RTPCR for COVID-19 out of which 24517 (8.24%) were diagnosed as COVID-19 positive. In contrast, 13614 (5.66%) patients were diagnosed as COVID-19 positive by RTPCR test done on a total of 240655 patients. It was observed that overall RTPCR positivity was higher in the second COVID-19 wave in comparison to the third wave and the difference in positivity was statistically significant as tabulated in Table 1.

**Table 1:** Characteristics of RTPCR results of COVID-19 samples in second and third waves of the pandemic RTPCR: Reverse transcription polymerase chain reaction, COVID-19: Coronavirus disease 2019

	Second Wave		Third Wave		P-value
Negative cases	267851	(90.02%)	225904	(93.87%)	Chi-Square = 3311 Degree of freedom = 3 P value <0.0001
Positive cases	24517	(8.24%)	13614	(5.66%)	
Repeated Sampling	5185	(1.74%)	1130	(0.47%)	
Equivocal	1	(0.0%)	7	(0.00%)	
Total	297554	(100%)	240655	(100%)	

The mean age of the COVID-19 RTPCR positive patients was (35.5 years vs. 37.4 years) years while the median age was (33 years vs. 35 years) in the third wave vs. the second wave respectively. It was observed that the proportion of positive cases in children ≤10 years in the second and the third waves were quite similar i.e. 3.59% and 3.40% respectively whereas the proportion of positive cases in adolescents (10-20 years) was higher (12.96%) in the third wave in contrast to the second wave (10.15%). On the other hand, the RTPCR-positive adults (41-60 years) were found to be less in proportion during the third wave (26.65%) in comparison to the second wave (29.50%). This difference in distribution was found to be statistically significant (p-value <0.05) as shown in Table 2.

**Table 2:** RTPCR positivity of COVID-19 in different age groups and sexes during second and third waves of the pandemic RTPCR: Reverse transcription polymerase chain reaction, COVID-19: Coronavirus disease 2019

Age Group	Second Wave						Third Wave						
	Male		Female		Total		Male		Female		Total		
	N	%	N	%	N	%	N	%	N	%	N	%	
Children (≤ 10 years)	535	3.49	344	3.75	879	3.59	262	3.02	201	4.08	463	3.40	Chi Square=145.9 Degrees of Freedom=4 p-value=<0.0000001
Adolescent (11-20 years)	1441	9.39	932	10.15	2373	9.68	1058	12.18	707	14.35	1765	12.96	
Young adults (21-40 years)	7588	49.47	4243	46.23	11831	48.26	4315	49.67	2449	49.72	6764	49.68	
Adults (41-60 years)	4436	28.9	2797	30.47	7233	29.50	2387	27.47	1241	25.19	3628	26.65	
Geriatric (>60 years)	1338	8.72	863	9.40	2201	8.98	666	7.67	328	6.66	994	7.30	
Total	15338	100	9179	100	24517	100	8688	100	4926	100	13614	100	
Sex*waves	Chi Square= 5.924, Degrees of Freedom= 1, p-value= 0.01493												
Male age group*wave	Chi Square= 56.59, Degrees of Freedom= 4 p-value= <0.0000001												
Female age group*wave	Chi Square= 117.6, Degrees of Freedom= 4 p-value= <0.0000001												

Moreover, the sex-wise distribution shows that the proportion of males among positive RTPCR cases was augmented significantly in the third wave as compared to the second wave. Among males, in the third wave proportion of adolescents and young adults increased and the proportion of other age groups was decreased in comparison to the second wave. This difference distribution was statistically significant. Among females, in the third wave the age group ≤40 years was more involved in comparison to the second wave whereas positivity was decreased in age groups more than 40 years. This difference was also found to be statistically significant as tabulated in Table 2. 

**Figure 1:**
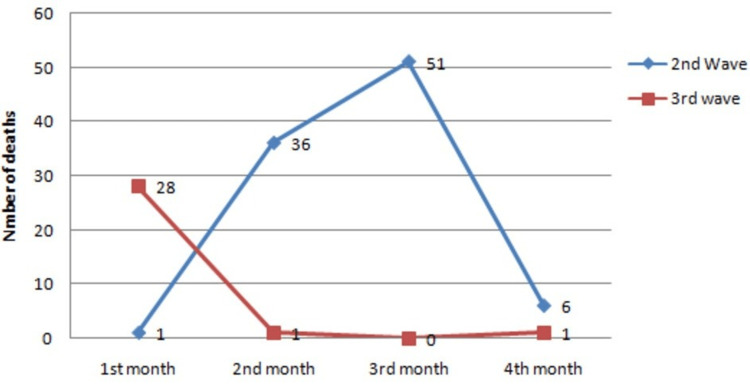
The death toll during four months of the second and third waves of the pandemic First month of the second wave = March 2021,                 First month of the third wave= January 2022 Second month of the second wave = April 2021,              Second month of the third wave= February 2022 Third month of the second wave = May 2021,                   Third month of the third wave= March 2022 Fourth^ ^month of the second wave = June 2021,                Fourth month of the third wave= April 2022

A total of 30 patients died of COVID-19 and other associated comorbidities during the four months of the third wave which is 1/3^rd ^lower than the patients (94) who died in the second wave during the four months of the study in the hospital which is statistically significant (p<0.01). The death toll during the two waves is depicted in Figure 1.

## Discussion

Our study encompasses the diagnosis of COVID-19 patients by RTPCR within the Meerut district in the second (March 2021 to June 2021) and the third (January 2022 to April 2022) waves of the COVID-19 pandemic. We observed in our study that 24517 (8.24%) of the total 297554 patients were diagnosed COVID-19 positive by RTPCR testing in the second wave. In contrast, 13614 (5.66%) patients were diagnosed COVID-19 positive by RTPCR testing in a total of 240655 patients during the third wave. A chief factor driving the second wave is the emergence of the more infectious variants of SARS-CoV-2, primarily the Alpha variant (B.1.1.7) and Delta variant (B.1.617.2) [12]. The positivity rate measures the proportion of people who test positive out of the total number of people who are tested. There is a significant decline in the overall positivity (of four months) from the second wave to the third wave which can be attributed to the fact that India started its vaccination programme on 16^th^ January 2021 which is still going on with booster doses and doses to 12-18 years of age as well. About 2,01,68,14,771 vaccine doses have been delivered till 23^rd^ July 2022 [13,14].

A low overall positivity in the third wave could be due to a vast majority of the cases remaining undetected, as a number of people with upper respiratory tract (including headache, cough, fever, generalized myalgia, and severe fatigue) infections were not getting themselves tested because of the milder nature of the disease caused by Omicron variant with relatively quick recovery time and very less hospitalization as stated in a study from Saudi Arabia [15].

The observed mean age of the COVID-19 RTPCR positive patients was 35.5 years and 37.4 years while the median age was found to be 33 years and 35 years in the third wave and the second wave respectively in the current study. There is a slight shift towards the younger age in relation to COVID-19 positivity from the second^ ^wave to the third^ ^wave as calculated through mean and median.

It was observed that the proportion of positive cases in children ≤10 years in the second and the third wave were quite similar i.e., 3.59% and 3.40% respectively. Before India braced the third wave of the COVID-19 pandemic, experts have raised concerns over its brunt on young children and adolescents owing to the fact that vaccines for children were unavailable and a higher number of children got affected in the third wave as per American data [16]. But the current study has disapproved the concern and found similar positivity in the second and the third waves among children below the age of 10 years. Our study shows that the proportion of positive cases in adolescents (11-20 years) was higher (12.96%) in the third wave in contrast to the second wave (10.15%) and the proportion of positive cases in different age groups (11-20,21-40, 41-60 years) was 87.44% in the second wave and 89.29% in the third wave and which is in line with the findings of a study from Kolkata which showed that patients in the age groups of 11-30 years, 31-45 years and 61-80 years were at high risk of COVID-19 infection in first and second waves [17]. However, earlier studies of epidemiology patterns found that the elderly will have the highest incidence of COVID-19, while those under 20 had low incidences of the infection [18,19]. Such inclinations towards the aforesaid age group can be due to the fact of their working fraternity with more human contact [20].

In the current study, the age group (41-60 years) has witnessed a significant (p<0.05) decline in the proportion of RTPCR positivity from the third wave to the second wave which is 29.50% to 26.65%. Similarly, the geriatric group (≥60 years) showed a decline from 8.98% to 7.30% which could be attributed to the factors that the vaccination started phase-wise; the first phase involved health workers and frontline workers [21] while the second phase involved people over the age of 60, residents between the ages of 45 and 60 with one or more qualifying comorbidities [22]. The other factor is that a large proportion got infected in the second wave and has developed protective antibodies.

In our analysis, the proportion of males among positive RTPCR cases was augmented significantly in the third wave as compared to the second wave. Among males in the third wave, the proportion of adolescents and young adults increased and the proportion of other age groups decreased in comparison to the second wave. This difference distribution was statistically significant. This is in line with the preliminary analysis by Tadiri et al. in 2020, which suggested that in countries with high gender inequality, men might be more likely to be exposed to COVID-19 as they are more likely to be employed [23]. Among females, in the third wave the age group ≤40 years was more involved in comparison to the second wave whereas positivity was decreased in age groups more than 40 years. A total of 30 patients died of COVID-19 and other associated comorbidities during the four months of the third wave which is 1/3^rd^ lower than the patients (94) who died in the second wave during the four months of the study in the hospital which is statistically significant (p<0.01).

Limitations

The current study has some limitations; firstly the data derived from the Uttar Pradesh (UP) COVID-19 Laboratory Portal database does not contain the clinical history of the patients. Various studies have reported that co-morbid factors like diabetes, cardiovascular disease, chronic renal disease, etc. have a role in mortality but we were not able to evaluate the same. Secondly, we have not included patients with RTPCR negative results but were showing the characteristics pattern symptomatically as well as radiologically. Lastly, the vaccine status of the patients was unknown as it had a big role in hospitalization as well as mortality.

## Conclusions

The current study indicates the importance of evolution in the mutant strains of SARS-CoV-2 causing the second and the third wave of the COVID-19 pandemic. The third wave showed low overall positivity (5.66%) as compared to the second wave (8.24%). Our analyses identified various variables like age groups (11-≥60 years) which differed significantly in the relative proportions of COVID-19 cases (RTPCR positive) in two waves but the age group (≤10 years) showed no significant difference in positivity. The two sexes also showed significant differences in positivity with predominant proportions of RTPCR positivity especially in males (21-60 years) while females less than 40 years showed high positivity. Considering the trends of the pandemic, India is still in danger of future waves of the COVID-19 pandemic but results from this study and ongoing vaccination programme would definitely encourage people not to panic and to adhere to COVID-19 appropriate behavior. Regular testing and surveillance with analysis of epidemiological and diagnostic data will help in the formulation of strategies to combat the COVID-19 pandemic.
